# *Love Carefully* and Without ‘Over-bearing Fears’: The Persuasive Power of Authenticity in Late 1980s British AIDS Education Material for Adolescents

**DOI:** 10.1093/shm/hkaa034

**Published:** 2020-09-27

**Authors:** Hannah J Elizabeth

**Affiliations:** Faculty of Public Health and Policy, Centre for History in Public Health, London School of Hygiene & Tropical Medicine, 15–17 Tavistock Place, London, WC1H 9SH, UK

**Keywords:** history of sex, history of emotion, HIV/AIDS in, Britain, history of education, adolescence

## Abstract

This article examines the 1987 British AIDS education leaflet *Love Carefully: Use a condom*, drawing on methodologies from both the history of emotion and literary analysis. The informative leaflet, produced collaboratively by the sexual health charities Brook and the Family Planning Association, was intended to prevent the spread of HIV among heterosexual adolescents, a group increasingly viewed as ‘at risk’ by adult producers of health education globally. Steeped in British teenage popular culture, it deployed an introduction from well-known teenage agony aunt Melanie McFadyean, a cartoon strip, and statements from celebrities. The cartoon offered a representation of the difficulties experienced by heterosexual teenagers negotiating the prospect of penetrative sex with a new partner, offering a successful example of condom negotiation, while sympathetically examining why some found condom use and AIDS difficult subjects to broach. The article argues the leaflet deployed emotions and authenticity to persuade teenagers to practise safer sex.

**Fig. 1:**
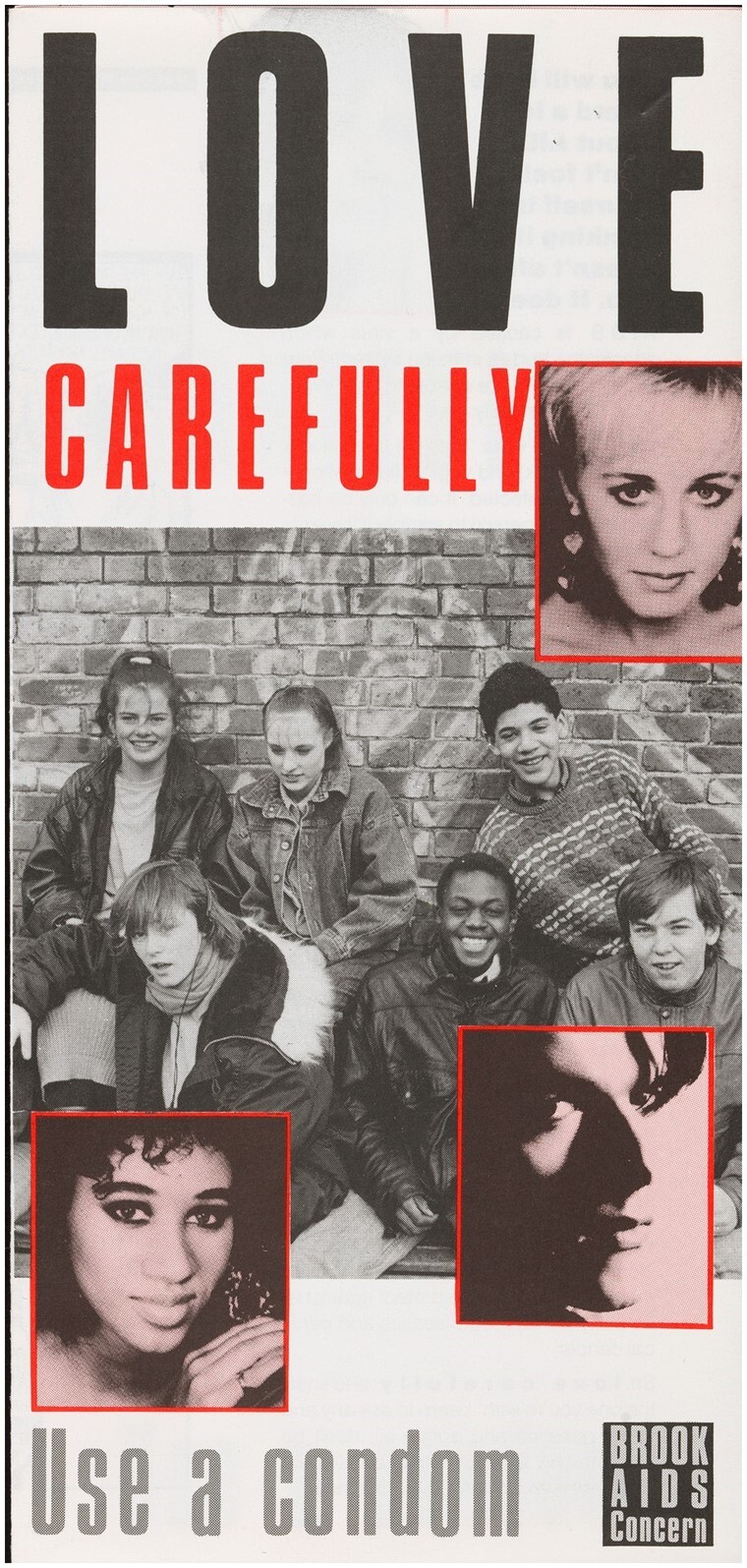
Front page of *Love Carefully—Use a condom*, first edition, 1987 (Fig. 1).^1^

In 1987, Brook,^2^ the British sexual health charity for young people, released the leaflet *Love Carefully—Use a condom* to encourage condom use among heterosexual teenagers. Deploying an introduction from well-known teenage magazines, agony aunt Melanie McFadyean, a cartoon strip, and statements from popular teen celebrities, the leaflet was intended to prevent the spread of HIV amongst heterosexual adolescents, a group increasingly viewed as ‘at risk’ by adult producers of health education nationally and internationally. McFadyean’s informative yet chatty prose introduced the idea that British adolescents were at risk from the ‘AIDS virus’, which could be carried in ‘blood, semen (spunk) and infected vaginal fluids’ but protected against by using a ‘condom (rubber, johnny, sheath, French letter)’.^3^ The cartoon strip, following Jim and Sylvia’s decision to practise safer sex, provided a representation of the difficulties experienced by teenagers negotiating the prospect of penetrative sex with a new partner within a heterosexual relationship. The cartoon’s script offered an ultimately successful example of condom negotiation between adolescents, while sympathetically examining why some young people found condoms and AIDS difficult subjects to broach, all while reinforcing the idea that safer-sex knowledge and practice were desirable aspects of British teenage identity.

The emergence of HIV in the early 1980s presented a new challenge for public health policymakers and front-line health service providers, particularly for those working in sexual health and education. While this moment of urgent policymaking has been the subject of intense scholarly scrutiny, much of this history has been written as an assessment or corrective against the charges of moral panic levelled at the New Right governments of the 1980s and 1990s. It has been argued that the New Right used AIDS as an opportunity to roll back social reforms and attack identity politics in favour of moral conservatism in response to media moral panics.^4^ Others have argued that AIDS policy and practice were shaped by a technocratic biomedical public health response, governed by informed elites rather than the vociferous politics of panic peddled by the press.^5^ The account offered here lies somewhere between these strands of scholarship, arguing that where the sex education and sexual health needs of adolescents were concerned, the policymaking response from government was a mixture of capitulation to populist demands, renewal of previous successful practice, and an adherence to advice from biomedical elites. Moreover, what education and advice was delivered to teenagers by non-governmental organisations (NGOs) was often formed in direct response to the ongoing panics fanned by the adult news media, institutional perceptions of teenage culture, and the individual agendas of service providers.

Launched at an orchestrated press conference on 15 April 1987 at Brook’s central London office, *Love Carefully* was celebrated by its producers for being the first education intervention of its kind.^6^ Brook claimed the leaflet, paid for by the Department for Health and Social Security (DHSS), represented a sophisticated foray into the sexual health education of adolescents. Visually reminiscent of the teenage magazines which formed a dominant arm of Britain’s youth media during this period,^7^*Love Carefully* deployed a combination of techniques to advocate condom use. The leaflet drew on celebrity endorsements, tropes from teenage magazine culture and Brook’s knowledge of adolescent sex and gender politics ‘to specifically address the problems young heterosexual people’ experienced when trying to follow advice ‘on how to protect each other from AIDS’.^8^ This was achieved, in part, by representing teenage experience as authentically as possible while positioning condom use as an act of love or care.

This article explores *Love Carefully* from its inception, production, reprint and reinvention, tracing what was new and old about the leaflet’s representation of AIDS and adolescence. It discusses how it, and its use, changed as the field of AIDS prevention in Britain rapidly evolved and the educational needs of teenagers were reassessed. In doing so, the article deploys the leaflet as a lens through which to explore broader histories of British AIDS representation to adolescents for the purpose of health education in the last decades of the twentieth century. This article adds to an existing body of HIV scholarship that draws on the cultural products associated with the virus to explore its social, cultural and emotional history. The article helps to redress two significant gaps in the literature: the absence of a history of HIV which engages with this disease’s cultural impact upon childhood and adolescence; and the limited scholarship on those non-governmental responses to HIV which specifically addressed sexually active heterosexuals. Though this case study furnishes us with a specifically British, rather than global, history of the virus’ cultural impacts, the methodologies deployed and attention to teen culture argued for here can be applied to cultural histories of AIDS in broader contexts. By drawing out the peculiarities of British youth culture imagined and addressed in *Love Carefully*, this article sketches how Brook constructed heterosexual youth in the era of AIDS. It demonstrates the fundamental importance of a history, which takes the specifics of age seriously, while adding to the literature on NGOs and communities that played pivotal roles in the early fight against AIDS.^9^

The article begins by outlining Brook’s place in the sexual health education of adolescents before the AIDS crisis. This sketches the parameters of the sex education arena, outlining which institutions and policies were influential in the early 1980s. The article then explores how AIDS education provisions were affected by the recognition that teenagers, as an emerging ‘at risk’ group, had specific sexual health education needs requiring dedicated materials and interventions. This locates Brook in its historical, cultural and political contexts, outlining Brook’s initial response to AIDS in relation to other emerging AIDS-related health education provisions for adolescents, its relationship with government and its ties to the Family Planning Association (FPA). The article then offers a close reading of both the production context of *Love Carefully* and the first edition of the text itself. Here techniques drawn from Bakhtin’s theories on intertextuality and dialogics are deployed to analyse the ideological and ‘work-like’ constituents of the text—those textual constructions that supplement ‘empirical reality by adding to, and subtracting from’ it.^10^ This methodology is chosen for its attention to the multiple ideological discourses disseminated by texts; those persuasive and emotive elements that are formed from, and in reference to, the cultural and emotional contexts of the text and its producers.^11^ Using an intertextual analysis to excavate the emotions behind and within the text highlights the key elements that mark the leaflet out as a representative amalgamation of non-governmental representations of AIDS to adolescents in this period. Furthermore, by combining intertextuality with an analysis of the emotional communities which produced this text, the article recovers what made *Love Carefully* a uniquely innovative and persuasive text, representative of Brook’s place in the wider sexual health education arena, and the institution’s feelings towards teenagers, their sexual health and their identities.

Brook was (and is) a prolific publisher of sex education materials for use with, and by, adolescents. Unfortunately, it is beyond the scope of this article to discuss the precursors to *Love Carefully*, nor will it be possible to discuss Brook’s later forays into AIDS education, except in those cases where *Love Carefully* was drawn on and redeployed. Similarly, while the article is careful to place this text in the context of the adolescent AIDS education policy and media that influenced its creation, and to which it was a constituent, it is not the intention of this article to revisit the policy or the material cultural history of AIDS, which has already been written.^12^

## Sex Education Before the AIDS Crisis

Awareness of AIDS in Britain emerged towards the end of 1981, with the first responses to the disease largely provided by voluntary communities of homosexual men who came together over the course of the 1980s to raise money for research, to provide support to loved ones, and to disseminate information about AIDS.^13^ Berridge argues that 1981–85 was a period characterised by self-help, which led to the formation of policy and expert communities such as clinicians, scientists, homosexuals and haemophiliacs.^14^ This evolving AIDS sector was part of the mixed welfare economy that emerged during the 1980s as greater emphasis was placed on community care. Community ‘self-help’ groups, activists and service providers were increasingly professionalised as the decade progressed and voluntary groups were called upon (and paid) to deliver a variety of services at the behest of the government. By the early 1990s, there were more than 500 voluntary agencies working within HIV/AIDS in the UK, competing for government money and attention.^15^ Organisations like Brook and the FPA must be counted among this number, fostering agency in their young audiences and service users in line with ‘self-help’ ideals while simultaneously lobbying on their behalf in the political spheres they could not access.^16^

The mid-1980s was also marked by AIDS’ transformation into a public health priority as its spread among heterosexuals was established and the virus isolated and identified. It is often suggested, in both lay and scholarly histories of HIV, that the advent of the AIDS crisis initiated increased openness around sex, especially in discussions with adolescents.^17^ Certainly, the threat of AIDS renewed the mandate for sexual health educators like Brook to talk openly about sex, but the influence AIDS alone had upon the conversation has been overemphasised, giving an impression of previous silence, and immediate change, following the emergence of AIDS. No such silence existed. Explicit discussions of sex were a regular feature in teenage magazines long before AIDS prompted the government to reassess the content of school-based sex education.^18^

Sex sold magazines and thus created opportunities for alliances between Brook, the FPA and sympathetic members of the magazine industry. Indeed, discussions of contraception and visits to Family Planning or Brook clinics formed regular features in *Just Seventeen* from its launch in 1983. Magazine feature writers and agony aunts took an active interest in the services provided by both the FPA and Brook, advertising their services and contact details whenever relevant.^19^ AIDS created a greater need for, and interest in, collaboration across the charitable and teenage media sectors and resulted in the setting up of several lobbying organisations devoted to AIDS education and sex education more generally.^20^ However, the vanguard of mass adolescent AIDS education was led by teenage magazines, who addressed AIDS in their pages from 1985.^21^ Consequently, when adolescent AIDS materials began to be produced outside the magazine sector, agony aunts like (FPA-trained) Tricia Kreitman of *MIZZ* magazine and Melanie McFadyean of *Just Seventeen* played pivotal roles. Agony aunts lent their expertise in sexual health promotion and teenage magazine production to organisations like Brook and the Health Education Authority (HEA), in addition to producing sexual education materials for the magazines themselves. Consequently, the HEA, recognising magazines’ unique ability to reach adolescents, placed health education adverts for five distinct AIDS education campaigns in several leading teenage magazines between 1988 and 1996.^22^

Beyond the pages of teenage magazines, childhood, education and teenage sexuality were repeatedly discussed within the public sphere. Sex education policy, and more broadly policy related to the rights and needs of the child,^23^ received a great deal of legislative attention during the 1980s and 1990s, with AIDS’ emergence affecting, but not instigating, interest in these topics.^24^

In part the proliferation of legislation around children’s rights and education was a result of, as Durham observes, the New Right’s willingness to opportunistically respond to ‘populist’ demands, which aligned with their intention to restructure the education system and critique the Left.^25^ This adherence to populist conservative politics was tempered occasionally under the Conservatives when ‘medical, health or scientific research was involved’, generating discordant policy decisions, such as the successful blocking of Victoria Gillick’s attempt to prevent under-sixteens from accessing contraceptive advice and rulings in support of embryo research following the Warnock Report, but also the passing of Section 28 and the 1993 Education Act, which both limited children’s access to sex education.^26^ These ongoing debates and the press interest, legislation and government-sanctioned guidance they generated form the backdrop and the parameters of Brook’s work in this period. Indeed, although the Gillick case was by no means unique,^27^ it is worth meditating upon it here as it legally outlined the limits of parental power, medical authority and adolescent agency.

Gillick brought legal proceedings against the DHSS and her Local Health Authority (LHA) in 1982 for refusing to promise to withhold confidential contraceptive advice or treatment to her underage daughters.^28^ Gillick aimed to establish that medical professionals, in providing contraceptive advice and treatment to children below the age of consent, were in breach of the 1956 Sexual Offences Act by acting as accessories to unlawful sexual intercourse.^29^ Additionally, Gillick argued, medical professionals could not give advice or treatment to her daughters without her explicit consent without being in breach of her parental rights.^30^

The courts ruled against Gillick in 1983, judging that medical professionals were neither in breach of the 1956 Act, nor did parental authority constitute ‘rights’ as they had no protection in law; rather parental authority engendered a responsibility.^31^ Unhappy with the judgment, Gillick appealed and successfully had the decision overturned in 1984, rendering the 1980 DHSS guidelines she had originally challenged illegal. Kenneth Clarke, then Secretary of State for Health, appealed to the House of Lords in a bid to reverse the Court of Appeals ruling. The House of Lords overturned the appeal court’s decision, placing medical authority above parental authority. It also moved the right to confidentiality and medical treatment back within reach of under-sixteens judged to have the capacity to consent—what would become known as Gillick Competence.^32^ Gillick Competence as an idea constructs the possibility of an agentic child, an actor able to make competent decisions within the specific geography of the medical setting or in the presence of a medical practitioner imbued with the power to assess (and therefore grant) agency. This agentic adolescent, able to seek contraceptive and medical advice, populated the audience Brook imagined for *Love Carefully*.

Outside the clinic and within school walls, legislation bolstered parents’ power, rather than children’s agency. For example, during the 1980s, school governance was restructured through both legislation and non-statutory circulars to ensure more parents were included on governing boards and to reinforce parental powers within the school system generally. Key among such legislation was the 1986 Education (No.2) Act, which transferred the authority to develop a sex education curriculum from LEAs to school governors.^33^ The Act allowed governors to dictate the content of sex education—if they allowed it at all—but mandated parental consultation within curriculum construction, made parent governors obligatory and allowed parents to withdraw their children from sex education lessons.^34^ The legislated threat of withdrawal, parental curriculum design powers and the limits on adolescent agency within school led to differences between sex education materials for use in and out of schools. In the case of Brook, this difference led to the creation of the Out-of-Schools Publication Group (OSPG).

Though it is important to consider how and what the government legislated around sex education and children’s rights, a wide variety of actors were able to influence children’s sex education in this period without legislative power. The government’s direct power was diluted by a structure, which had governed sex education in Britain since the 1960s, characterised by a ‘series of sub-contracting operations’, wherein NGOs were paid by government to deliver sex education.^35^ This allowed the government to respond to public pressure when necessary by providing or withdrawing funding but did not impose public consultation or parliamentary oversight for programmes’ philosophy, ideology or content.^36^ While the introduction of the National Curriculum in 1988 placed limits on teachers’ freedom,^37^ the late 1980s and early 1990s were largely marked by legislative decisions to reduce the government’s power in the classroom, handing it instead to parents.

In Britain, in the latter half of the twentieth century, formalised sex education remained firmly cast, as it was from its Victorian inception, as ‘a strategy of damage limitation’, the focus fixed on preventing unwanted pregnancies, moral decline, disease or loss of social status, rather than the possibilities of ‘pleasure and empowered choice’.^38^ Hall observes that herein lies the ‘dialectical struggle’ at the centre of conflicts over the necessity for, and aims of, sex education: those on one side ‘advocating the provision of clean, healthy scientific knowledge (by the standards of the day) to combat the sordid or partial information’ children inevitably picked up, thus promoting healthy risk-free behaviours.^39^ And those on the other side proceeding from the presumption that the act of educating children about sex ‘was to corrupt primal innocence’, encouraging the manifestation of sexuality and sexual behaviours that might otherwise not develop.^40^ As Elizabeth argues, the child thus constructed is ‘a corruptible innocent that cannot be in any way sexual and is annihilated through inappropriate education; sexual knowledge transporting the ignorant/innocent child out of childhood and into knowing adulthood, or worse still, into a vulnerable hinterland of adolescence’.^41^ This vision of childhood is one marked by pessimism and nostalgia, the advent of agency through adolescence or adulthood figured as a marker of childhood’s end. More optimistic visions of the child, imbued with rights, afforded limited agency, imagine a child who can (and therefore should) be protected through sex education from the risks associated with sexuality. Legislation regarding the rights of children and adolescents, and their sex education, was marked by these dissonant ideas, united by an overall attempt to govern the unruly bodies of the young, optimistically guarding the future against calamity or pessimistically defending the status quo against the ever-encroaching threat of permissiveness.

I use the terms optimism and pessimism here to draw attention to the emotional difference that lay behind these disparate visions of the child and childhood: visions of the child as an agentic figure are imbued with hope for the future; those imagining a vulnerable innocent steeped in nostalgia, viewing the future as fearful. These constructions of the child were both generative and indicative of the ideals of adults existing within particular emotional communities. Emotional ties to some degree account for productive relationships between institutions, despite varied levels of judicial and legislative responsibility and power, and in some cases, near-constant clashes over territory and jurisdiction. Here I draw on Barbara Rosenwein’s concept of the ‘emotional community’ to guide my analysis. Analogous to ‘social communities’, their emotional nature is discovered through the attention of the researcher to the ‘systems of feeling’ that govern them. These systems amount to shared definitions of what individual members and the collective community ‘define and assess as valuable or harmful to them’; assumptions about the emotions of others outside the community; ‘the nature of the affective bonds between people that they recognize; and the modes of emotional expression that they expect, encourage, tolerate, and deplore’.^42^ Here my attention is most acutely drawn to the ‘affective bonds’ between sex education producers and their shared assumptions about the emotional communities that existed outside their own—namely those formed by the adolescents they hoped to persuade to use condoms and the sex education policymakers and funders within government whose chagrin they wished to avoid.

To excavate these ‘systems of feelings’, I draw on Lacanian literary critique, which maintains that the creation of a text such as *Love Carefully* begins first with the imagining of an audience by the text’s creator(s); the situated but imagined receiver(s) reacting to context and text according to the hopes and fears of the author(s). Reading authorial intent, ideology and emotion off a text like *Love Carefully* by excavating the audience imagined by it in this manner is a fruitful undertaking, rendering the sub-textual normative narratives of childhood and sexuality underlying such texts available to scrutiny. *Love Carefully* was a text representative of a particular emotional community; the creation of it a political act born of the desires of one emotional community—Brook and FPA—to intervene in their vision of an emotional community of teenagers. An attempt to intercede in the lives of an imagined audience presumed to be in possession of particular emotional dispositions and governed by certain emotional regimes and consequent behaviours—in this case, the use or rejection of condoms.

Approaches that borrow from Lacan, as this one does, can suffer from a degree of myopia, placing authorial intent and emotion in view while losing sight of the audience as a living, varied, fluctuating, agentic and fractured object. The audience is an entity whose context, emotions and experiences render them as much the creator of the text’s meaning as the original producers. An attention to intertextuality—the referential nature of texts, their dialogic quality and their ‘work-like’ aspects—foregrounds the primacy of prior audience knowledge, emotion and context in shaping the reception and meaning of a text.^43^ By examining the referential, dialogic and persuasive elements within a text, the underlying emotion, intent and imagination of producer and audience detectable in the narratives, ideologies and discourses framed by the text become available to scrutiny. Consequently, an intertextual approach provides a useful bridge between the social and cultural contexts of the emotional communities that authored the text—Brook and the FPA—the imagined audience of the text itself—here a variety of teenage emotional communities—and finally the situated teenagers who actually viewed the text. Though the reception of *Love Carefully* among teenagers is not the subject of this article, an awareness of the dialogic and intertextual nature of texts, and so their relation to the ‘ongoing social and political transformations’ that produce and give them meaning, is vital.^44^ Used in combination with the consideration of emotion suggested by Rosenwein, and the attention to authorial intent advocated by Lacan, an intertextual analysis renders the persuasive or work-like elements of the text and the situated emotional communities that produced them open to observation.

## Brook Before AIDS

The first Brook centre was opened in London in 1964 by its founder Helen Brook. Two years later, the Birmingham Brook centre was opened, acting as a base in the Midlands but also providing the location where the Education and Publications Unit would later operate.^45^ The stated aim of Brook was ‘the prevention and mitigation of the suffering caused by unwanted pregnancy by educating young people in matters of sex and contraception and developing in them a sense of responsibility in regard to sexual behaviour’.^46^ Within the structure of Brook, the main function of the Education and Publications Unit was ‘to produce and distribute nationally the Brook catalogue and education and information material’.^47^ By the beginning of the 1980s, Brook was a substantial institution providing sexual health services to the under-25s in a number of major cities across Britain. The organisation was funded on a national level via ‘a grant from the DHSS, contributions from Branches and donations’, while branches were ‘funded at a local level by the NHS, social services, sales and client donations’.^48^ This funding structure placed Brook under a degree of governmental oversight. For instance, the funding offered by the DHSS to Brook in 1981, some £30,000 for 3 years, was conditional on Brook consulting ‘more widely with educational interests suggested … by the Department of Education and Science’ and seeking approval for ‘any draft material intended for use in schools’.^49^ This condition led to the founding in 1982 of the School Publications Advisory Panel (SPAP) under the guidance of Department of Education and Science (DES) representative Jasper Ungoed Thomas.^50^ Membership on the SPAP required DES and Brook Advisory Committee (BAC) approval and marked a direct line of government influence in the running of the educational arm of Brook’s operations.^51^ The DES’ power over Brook operations and publications stopped at the school gate and was limited by a division of labour within Brook’s Education and Publication Unit between publications intended for use in and out of schools. This allowed the Out-of-Schools Publication Group (OSPG), the organisation within Brook responsible for the creation of *Love Carefully* alongside Brook AIDS Concern, to enjoy more freedom. Consequently, OSPG largely avoided embroilment in the territorial clashes, which marked DES dealings with other NGOs operating in schools, and it was less restricted by the evolving obstructive education policies of the 1980s and 1990s.

However, while the OSPG was influenced by, rather than subject to, the rules governing school-based sex education, the group was limited by the requirement to differentiate its work from the FPA’s Publication Unit, lest Brook and the FPA lose Department of Health (DoH) funding due to perceived duplication of services.^52^ This led the two organisations to informally divide the sex education field between themselves; ‘Brook would specialise in young people with special needs’, while the FPA ‘would continue to produce all materials on family planning for a mainstream audience’.^53^ This division of labour was ensured by quarterly meetings between Brook’s General Secretary and Press Officers and those in the equivalent positions within the FPA ‘to share publication plans’.^54^ Indeed, when the OSPG was set up on 11 July 1986,^55^ Helen Martins, the FPA’s Publications Officer, was asked to be a founding member.^56^ The advent of the AIDS crisis saw both institutions expand their repertoires, reaching broader audiences and inevitably stepping on each other’s toes despite maintained channels of communication and shared membership to several AIDS education bodies.^57^ This complicated though predominantly productive relationship between Brook and the FPA would have a direct effect upon the content of *Love Carefully*, a leaflet initially paid for and produced at the behest of the DHSS.^58^

## Recognising the Need for AIDS Education Targeting Adolescents in a Hostile Policy Arena

The need for a specific response to AIDS intended for the consumption of heterosexual adolescents was recognised by front-line Brook workers who were increasingly ‘finding that young people […] picked up a lot of information about AIDS, but […] developed over-bearing fears and worries about the disease’.^59^ Brook was not alone in recognising the need to ‘myth bust’ and reassure adolescents about the dangers posed by AIDS, but like many institutions, this realisation seemed to occur rather late, in 1987. While teenage magazines addressed AIDS myths in their pages from 1985 onwards, institutions like the HEA, the British Medical Association (BMA), the FPA and Brook would not launch AIDS-related materials specifically for the consumption of adolescents until 1987. Indeed, while policymakers and NGOs struggled to produce coherent legislative responses to the AIDS crisis targeted at teenagers, British teenage magazines were busy promoting knowledge of safer sex as a valuable aspect of modern teenage femininity—the provision of safer-sex information becoming one of the many ways teenage magazines sold.^60^

By 1987 limited consensus about the AIDS education needs of teenagers had evolved, with themes such as myth busting, dismissing bigotry, consent, and condom negotiation appearing across a variety of teenage media, including texts produced by the government. This was in part the result of the collaborative forum in which AIDS policy was written, with organisations like the HEA, BMA and FPA sharing key members and borrowing policy and practice from one another as they attempted to navigate the ideological minefield of sex education and children’s rights without aggravating the DES.

The idea of AIDS ‘myths’ reoccurs across both the internal archives of institutions involved in the public health campaigns against AIDS and as an explicitly addressed problem in the materials produced for adults and adolescents.^61^ Indeed, myth busting was one explicit goal, among others, of the HEA’s 1987 AIDS strategy.^62^ The HEA strategy also argued ‘adequate information for young people in full time education’ and ‘16–24 year olds not in schools’ must be made a priority—though what was meant by ‘adequate’ here was left open to individual education providers.^63^

Ambiguity gave those providing education to adolescents a degree of wriggle-room, allowing them to follow their own agendas. Those who pessimistically felt that sex education was a corrupting influence were left able to provide the bare minimum within curriculum parameters. Meanwhile, more optimistic educators argued for, and produced, more explicit information, believing it held greater protective or empowering potential. This resulted in wide variations in the AIDS education of adolescents in Britain across the late twentieth century, both within and outside the formal education system.^64^ While the introduction of the National Curriculum in 1988 made some limited biological aspects of sex education compulsory, AIDS did not become part of the statutory sex education curriculum until 1991.^65^ It was removed some 2 years later, along with other aspects of sex education such as contraception, with the introduction of the 1993 Education Bill.^66^ The variations this propagated were rendered starker still by another aspect of the 1993 Education Bill; the parental right to withdraw children from non-statutory sex education lessons.^67^ It was in the context of this disjointed, contested and fast-changing arena of sex education legislation and provision that *Love Carefully* was produced, disseminated, reprinted and repurposed.

## ‘It is Simpler to Talk About AIDS, But it Creates Misinformation’^68^: Medical Accuracy versus Clarity of Message in the Collaborative Production of *Love Carefully*

As the 1987 press release for *Love Carefully* explained, ‘By suggesting ways of raising the AIDS issue and discussing condom use with their partners’, the leaflet aimed ‘to help young people, particularly young women, develop the assertion skills they need to protect themselves from AIDS.’^69^ This was achieved textually through numerous strategies that belie the productive influence of existing and developing collaborative relationships between Brook, the FPA, the Terrence Higgins Trust and key teenage magazines. Examining this collaborative production process sheds light on what aims were prioritised within the text, which strategies were assumed to be persuasive and, ultimately, how NGOs like the FPA and Brook imagined the needs, emotions, and lives of teenagers.

Pressed by the DHSS to produce an AIDS education leaflet targeting heterosexual adolescents quickly, Brook ‘by-passed the normal committee consultation’ in deference to ‘the urgency of the situation’, receiving speedy approval for *Love Carefully* from the DHSS and Chief Medical Officer and ‘a grant from the DHSS’ to fund printing.^70^ Despite circumnavigating the usual internal processes, Brook still sought to consult the FPA. They requested comments on two drafts of *Love Carefully* from interested members of both charities and later met with the FPA’s head of publications, Helen Martins, and her team to consult directly.^71^ This swift consultation—*Love Carefully* was launched on 9 April 1987, less than 3 months after the FPA was first asked for input—was facilitated by ties between the publication arms of Brook and the FPA made necessary by earlier DHSS stipulations regarding duplication of services.^72^ Indeed, concerns over issues of jurisdiction and duplication framed much of the discussion between the two NGOs about the production and dissemination of *Love Carefully* throughout the 1980s and 1990s. Agreements over what should be done about AIDS quickly led to more fraught considerations of how shared aims should be delivered on, by whom, where and when. Indeed, as will now be discussed, precursors to later disagreements and concerns are detectable in the ultimately fruitful collaborative production of the leaflet.

**Fig. 2:**
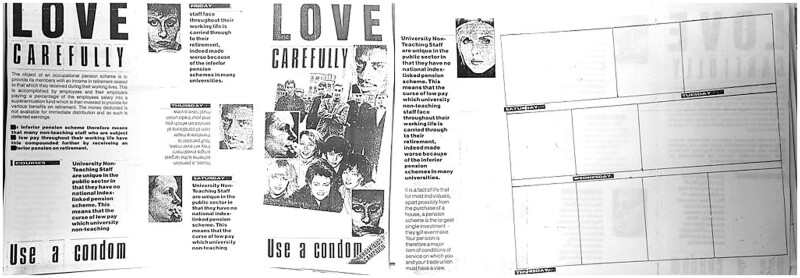
*Love Carefully* draft 1 layout photocopy, 21 January 1987 (Fig. 2).^73^

The FPA’s AIDS Working Group (AWG) were sent photocopies of Melanie McFadyean’s introduction, cartoon strip script and the proposed layout for *Love Carefully* in late January 1987. The first round of comments from the FPA’s AWG included contributions from FPA medical officers and Alastair Service, the FPA General Secretary, former Vice-Chair of the dissolved Health Education Council and the then Vice Chairman of the HEA.^74^ While feedback was mainly positive, there were a few criticisms. Most commonly, annotations were made regarding clarity of message, presence and accuracy of the latest medical information, how persuasive particular elements of the leaflet were likely to be, and what need not be included because the FPA would provide it in their own materials. There were also more subjective complaints about the tone of the cartoon script, the saccharine elements garnering the annotation ‘yuk!’ from one commentator.^75^ Many annotations suggested adding additional explicit information about the risks engendered by specific sex acts such as oral sex. Such additions would shift the focus of the leaflet from explicating condom negotiation between a heterosexual teenage couple to a broader more inclusive narrative about safer sex between consenting partners. While this expansive narrative did not survive the editing stage, it is worth noting that the desire existed among the sexual health educators of the FPA and Brook to provide explicit, accurate and broad representations of sex and sexuality. This unsatisfied wish for inclusivity forms the backdrop of frustration underlying many of the comments examined below. Indeed, what was left out of the published edition holds as much significance as what made it to print. As Crowther points out, agnotology—the study of culturally produced ignorance—is fundamental to understanding how education and entertainment media may both promote and maintain ignorance as well as knowledge.^76^

Concerns expressed by Service regarding what information was present and absent in the first draft of McFadyean’s introduction acknowledge the powerful effect of constructed ignorance. Service complained it ‘says very little about how disease works & what it is’ and wondered charitably ‘is this deliberate to keep it short?’ In a similar but more critical vein, Toni Belfield, a medical information officer for the FPA, argued for greater ‘educational input’, feeling it was vital to make clear ‘you can’t catch AIDS. You get HIV which might or might not lead to AIDS eventually. It is simpler to talk about AIDS, but creates misinformation’ (emphasis in original).^77^ It was a shared assumption among those creating and editing *Love Carefully* that simpler messages would carry greater persuasive force. However, so too were worries about what meaning might be lost, or worse misapprehended, in seeking this simplicity. Indeed, Belfield later repeated ‘you can’t catch AIDS’ in reference to *Love Carefully’s* cartoon script, again with emphasis.^78^ This highlights a perpetual anxiety felt by those producing AIDS literature for the consumption of adolescents (and adults)—that misinterpretation of texts might perpetuate, rather than disrupt, the myths that circulated around AIDS. Producers thus imagined their audiences as eager for knowledge but vulnerable to fear and confusion.

Similarly, *Love Carefully’*s producers were concerned the text might make teenagers more fearful by normalising anxieties through depicting them in the cartoon. The cartoon script, which changed little between its first draft and publication, opens with Sylvia discussing both her desire to have sex with Jim and her fear of AIDS with her friend Jane. Jane responds by suggesting they use condoms. Following Jane’s advice, in the next scene, Sylvia broaches condom use and her AIDS fears with Jim. He reacts badly, exclaiming ‘Are you suggesting I’ve got AIDS?’. Sylvia hastily responds ‘No! It’s just that everyone is at risk now, even you and me. We have to use condoms; they help stop the AIDS virus being passed on during sex’. Jim responds dismissively ‘But I couldn’t use one of those things’. While thinking to himself ‘Maybe she’s got a point’. Lines like ‘It’s just everyone is at risk now, even you and me!’ regarding AIDS and ‘But I couldn’t use one of those things. Nobody uses those things anymore!’ regarding condoms attracted criticism. Both these lines were objected to not only on the grounds of accuracy but also in reference to the emotions they would likely elicit in the intended audience. To the idea that ‘everyone is at risk’, one commentator objected ‘no this is not so’, while another queried ‘might this scare people more than is justified?’.^79^ This reaction would counteract one aim of the leaflet—to reduce the ‘over-bearing fears and worries about the disease’ felt by teens.^80^ Objections to the representation of distaste regarding condom use were stronger still, arguing the above lines ‘perpetuate the myth that the condom is awful!’, and that it was ‘wrong to generalise’ that ‘nobody uses those things’ as ‘– stats do not bear this out in UK’.^81^

Herein lay a tension; through authentically depicting familiar teenage anxieties, producers hoped to make the text salient and to address teenage fears, offering a realistic representation of condom negotiation through the cartoon script. But it was also felt that if enough space were not given to vital discussions of condom use, negotiation, and AIDS in the cartoon, then representations of these fears might potentially normalise myths. It was feared that by authentically portraying the mistaken attitudes towards AIDS and condoms circulating within teenage sexual culture, such attitudes would be proliferated. Belfield, annotating the first draft, wrote ‘The script seems to only focus on use of condoms, there is nothing on communication, yet the title suggests talk more’, Belfield repeated these thoughts later with a less expansive ‘how about suggesting more communication?’^82^ Such criticism, of a script where condoms and AIDS are discussed and ultimately the decision to practise safer sex taken, might seem unjust, but upon closer examination, these charges stand. The fear that everyone was at equal risk of ‘AIDS’ is left wholly unmitigated by the cartoon’s narrative, and while distaste towards condoms is challenged, it is done so in a limited and specifically (binary) gendered way; Sylvia and Jim discuss their condom-related anxieties with friends of the same sex, rather than one another.

## ‘It’s the Real Man Thing to Do’: Gendered Condom Use

**Fig. 3:**
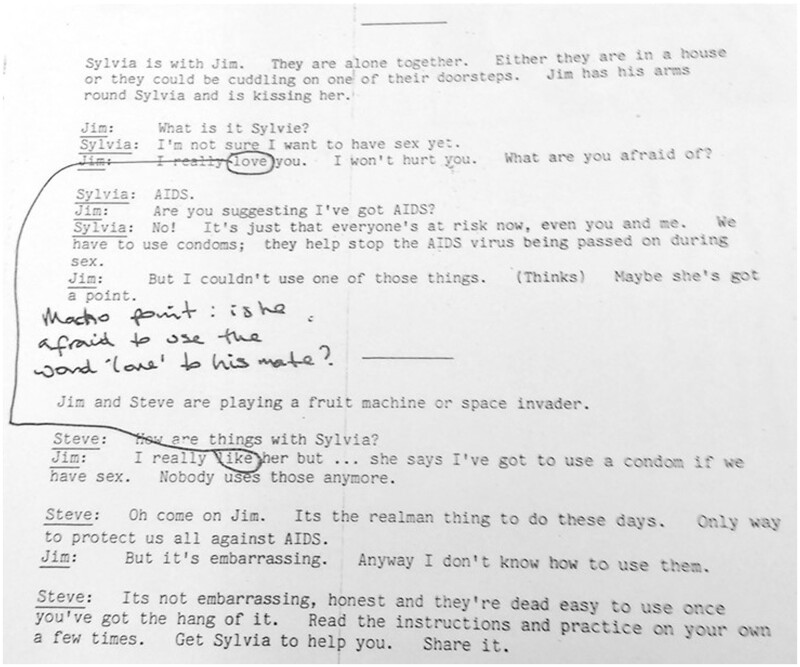
‘Macho point: is he afraid to use the word “love” with his mate?’ Annotated photocopy of draft 2.^89^

While ultimately Jim and Sylvia decide they will use a condom if they have sex, this decision takes place with their friends, rather than one another. When Sylvia requests the use of condoms during sex, Jim initially dismisses it, then goes on to discuss condoms with his friend Steve, while Sylvia discusses their use with her friend Jane. Jane and Steve are having safer sex together and so share the knowledge of their sexual relationship and condom use with their friends, but separately, rather than as a group despite this mutual friendship. In the end, it is not Sylvia who convinces Jim to use a condom in the future, but Steve, who appeals to Jim’s sense of masculinity by arguing condom use is ‘the real man thing to do these days’ to persuade him (Figure 3).^83^

In contrast, when Sylvia explains to Jane ‘Jim doesn’t want to use condoms but I’m not going to have sex with him till he agrees. It’s the only way to be safe’, Jane responds by framing condom use as an act of care—‘He will if he cares enough for you’—not dependent on gender and suggests Sylvia take responsibility for the provision of condoms: ‘You’d better carry a packet of condoms with you just in case’. To this Sylvia responds ‘But he’ll think I’m sleeping around’, so Jane attempts to reassure her, repeating the idea that it is an act of ‘care’ with ‘Well you know that’s not true. You’re just taking care (emphasis in original).’ This exchange, presenting the responsibility for condom use as shared while sympathetically acknowledging teenage girls’ fears about accusations of promiscuity, is fairly typical of adolescent entertainment media’s representations of condom negotiations. While the DHSS and HEA would favour narratives that accused teenage girls who did not practise safer sex of cowardice and complacency, running adverts with taglines like ‘AIDS – You’re as safe as you want to be’ across the late 1980s, teenage magazines took a more feminist track from as early as 1983.^84^ Teenage magazines blamed systemic sexism for contraceptive failures and contextualised the disproportionate contraceptive responsibility teenage girls carried largely through narratives of masculine irresponsibility. Specifically, magazines critiqued the behaviour of individuals or the patriarchy as a whole, portraying idealised teenage boys as struggling to overcome misogynistic and outdated gender politics in favour of more caring and egalitarian performances of masculinity, which were receptive to the confident teenage feminine identities sold by the magazines. These critiques adhered to a sex-related binary conception of gender and were carefully written to avoid alienating male readers, a small but valuable minority of teen magazines’ audience. Similarly Brook, in an attempt to entice male and female teenagers to use condoms, presented a more positive view of both genders’ sexuality than the HEA or DHSS adverts. Connecting ‘care’ and ‘love’ with teenage sexuality and being ‘manly’, the leaflet acknowledges the weight of teenage feelings, giving them credence, while promoting the idea that sex was better, and safer, in the context of mutually shared feelings and responsible behaviour.

Here it is worth unpacking the intent behind the deployment of the concepts of ‘love’ and ‘care’—or loving carefully—in the context of adolescent sexuality and public health education. Positioning condom use as an act of care or love is a reoccurring technique in safer-sex and HIV/AIDS health education initiatives. While the Terrence Higgins Trust and Gay Men Fighting AIDS attempted to normalise condom use between men through eroticising safer sex in their health promotion materials, as well as associating it with care,^85^ adverts targeting heterosexual teenagers (and to a lesser extent adults) tended to emphasise safer sex as love-seeking behaviour—positioning condom use as a demonstration of care aligned with the pursuit of an eventual loving monogamous relationship. Moreover, to delay the onset of first sex and discourage promiscuity, sex was presented to teenagers as something that could *only* be achieved safely within a loving relationship and with the ‘right’ person who had to be sought. This was achieved by presenting love as a rare but vital component of safer sex, teenage first-sex as deeply meaningful, and by repeatedly constructing condom use as a care act.

Despite this scene that addressed the fears many young women felt around condom use, the more dominant narrative of condoms as distasteful and somehow connected to masculinity caused consternation among reviewers of the first draft. Indeed, Helen Martins, the FPA’s publications officer, questioned the framing of condom use within the leaflet as a whole, asking ‘will young women feel they are equally a target audience for this …’. The use of appeals to masculinity garnered further criticism later when the FPA flagged similarities between *Love Carefully* and their ongoing Men Too campaign,^86^ feeling that encouraging heterosexual men to use condoms was moving into their area of expertise.^87^

When photocopies of the second draft of *Love Carefully’*s introduction and cartoon script were circulated, this time with the accompanying images, FPA commentators framed their annotations with a chastisement ‘Please see all the general and specific comments made on earlier draft: most of these still apply, so have not been repeated here’.^88^ Some comments were repeated, however, indicating the importance of the sentiments they expressed, and frustration with changes not yet made. Again, masculinity became a focus of discussion, one commentator questioning Jim’s expostulation of ‘love’ to Sylvia, which he later softens to ‘like’ when expressing his feelings in conversation with Steve (Figure 3).

**Fig. 4:**
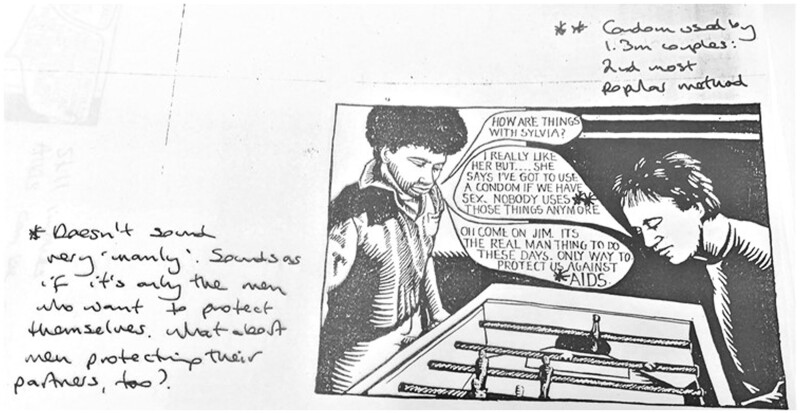
‘Doesn’t sound very “manly”’: annotated photocopy of *Love Carefully* cartoon draft 2.^91^

Similar expressions of distaste frame the suggested image that was proposed to accompany this scene (Figure 4); the annotated draft reads ‘Doesn’t sound very ‘manly’. Sounds as if it’s only the men who want to protect themselves. What about men protecting their partners, too?’^90^

Both these lines were changed in the final published version of the cartoon. Jim expressing his ‘love’ (rather than ‘like’) for Sylvia in conversation with Steve, and Steve saying condom use is the only way ‘to protect us all’ rather than merely ‘us’ (Figure 6). These changes might seem subtle, but together they work normatively to emphasise the gravity of the emotional and physical risk inherent in a teenage sexual encounter and present an idealised relationship and condom negotiation outcome. Consequently, loving rather than liking a partner becomes as much a prerequisite for a caring sexual encounter as the universalised use of the condom, which will ‘protect us all’. The use of a condom itself becoming an act of love or care. This narrative, which suggested love had a protective quality and condom use was akin to a care act, was a common component of teenage magazines discussions of sex, where it was often suggested that first sex should only happen with ‘someone you love’. Such discussions were also accompanied by explorations of the myriad difficulties encountered by young women trying to persuade young men to take contraceptive responsibility.^92^

## Communities of Shared Sarcastic Humour

Despite ongoing tensions regarding who should deliver AIDS education to teenagers, particularly where condom use was central in the text, relations between Brook and the FPA were dominated by shared goals, similar visions of teenage identity and mutual respect. This shared vision of the world, and how to change it, is at its most obvious in the traces of humour, which can be found in the archive. It is detectable in the humorous letters sent between Brook and FPA members of the OSPG^93^ and the sarcastic annotations that clutter the first and second drafts of *Love Carefully*, especially those making reference to the tone of the cartoon, particularly the proposed final lines. The first draft of the cartoon ended with Sylvia’s enthusiastic final line ‘Oh that’s great Jim, you really do care’ in response to Jim’s agreement to use a condom. To which it was proposed, he brightly respond ‘Nothing’s going to spoil this romance’. Service, pulling no punches in the knowledge that his response would be understood as having humorous intent, underlined this final line and wrote ‘Yuk!’, while another commentator sarcastically wrote ‘Strong stuff!’. We can read sarcasm and humour off the page here by placing these directionless comments in the context of the more sensible actionable criticism discussed earlier, which offered suggestions for additions or changes, drawing attention to elements of the text, which might be problematic or misconstrued.^94^

It is significant that the lines ‘Oh that’s great Jim, you really do care’ and the responding ‘Nothing’s going to spoil this romance’ were read as too saccharine by commenters. The implication behind the comment directing the producers behind *Love Carefully* to imagine a discerning teenage reader finding the line equally sickly and consequently ignoring the safer-sex message in response to the insincerity of the final exchange. In the second draft, an amendment was proposed, ending Sylvia’s line ‘Oh that’s great Jim you really do care. Till then, there’s always kissing and hugging and stroking … ’, which was responded to by one commenter with ‘bit twee?’. The final draft of the cartoon had the line return to the original ‘Yuk!’ formation, the ‘twee’ suggestions of other pleasure-seeking non-penetrative sexual activities left out.

This shared sense of humour demonstrates an empathetic relationship between the two organisations, the existence of an emotional community of adults organised around a shared desire to take the needs and experiences of adolescents seriously. The sarcasm and silliness here acts to chastise ‘insincerity, pomposity, stupidity’,^95^ drawing attention both to *Love Carefully’*s shortcomings as a text and to the wider cultural context, which prevented those involved in the sex education of teenagers from delivering texts that had bite. Jokes, even those in-jokes made within an intimate closed circle such as that between Brook’s OSPG and the FPA’s AWG, hold significance beyond the indication of shared humour or the existence of an emotional community. As Mary Douglas argues, jokes are ‘a victorious tilting of uncontrol against control, … the levelling of hierarchy, the triumph of intimacy over formality, of unofficial values over official ones’.^96^ Jokes’ presence in the archive are a jovial marking of Brook and the FPA as wilfully *other*. Brook and the FPA’s shared jokes established them as a group of like-minded peers battling the sex education policy, which would silence their efforts and refute the ideals that they based their interventions on –teenagers as agents, sexual knowledge as a good.

The significance of this buoyant emotional community of AIDS educators, intent on the empowerment of adolescents lies in the ways it differs from other communities of AIDS education producers. Those who saw children and adolescents as innocents in need of protection viewed the threat of AIDS with some combination of panic over the potential spread of the disease, and anxiety over the potentially damaging effects of providing sex education to the uninitiated. Theirs was a community of pessimism and reluctance; viewing the sex education of adolescents as at worst a damaging incursion upon the rights of parents and innocence of children, and at best a necessary evil (rather than liberating good). While the DES blocked attempts to include explicit content in school materials and the BMA produced an AIDS board game that avoided discussing sex,^97^ the FPA and Brook were suggesting the addition of more explicit details and worrying their materials would be read as inauthentic by their discerning teenage readers.

Despite shared ideals, not all criticisms were taken on board, indeed many aspects of the first draft that had caused consternation made it into the final draft. For instance, the term ‘AIDS virus’ rather than ‘HIV’ was used with little clarification despite being flagged several times for its inaccuracy and the narrative of inevitable decline towards AIDS it implied. Here the clarity of the message ‘use a condom’ was favoured over the opportunity to clarify the trajectory of HIV. This change, from AIDS to HIV, would only be made in the second edition of *Love Carefully.*

**Fig. 5:**
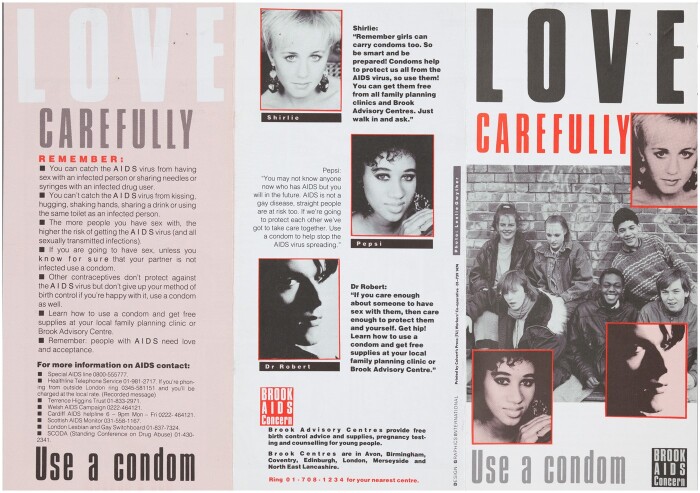
*Love Carefully—Use a condom*, first edition, front and back covers (Fig. 5).^98^

## Reading and Using Love Carefully: Dissemination and Repurposing

While changes in tone and language were made during the drafting process, the cartoon text maintained its gendered narrative of male reluctance to use condoms and female fear of AIDS. Moreover, the leaflet only mentioned HIV transmission between men and women despite early suggestions for a more inclusive introduction that would discuss same-sex relationships. Moreover, anal sex, which was included in the first and second draft of the introduction, was omitted from the final published edition of *Love Carefully.* Oral sex, and the risks it might potentially carry, also went unexamined despite requests on draft two for its inclusion. Given these omissions and shortcomings, we might ask if the triumphant press release produced for *Love Carefully* can really make the claim that the leaflet adequately suggested ‘ways of raising the AIDS issue and discussing condom use’? Especially we might question if its aim to ‘help young people, particularly young women, develop the assertion skills’ was fulfilled? As Belfield asked, would young women ‘feel they are equally a target audience’ for the finished product?^99^

**Fig. 6:**
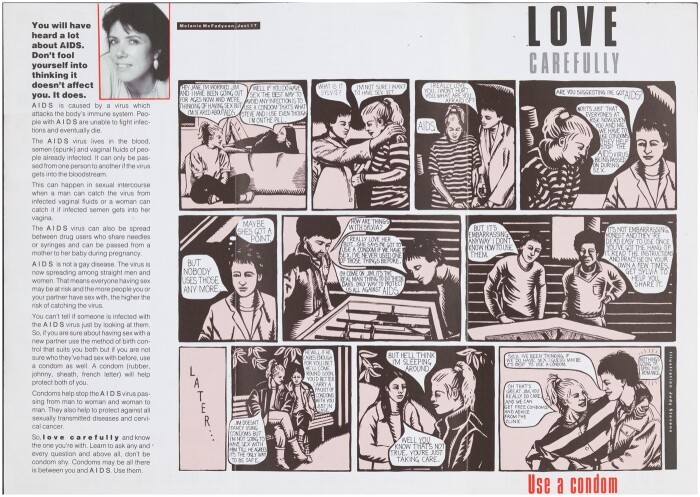
‘It’s the real man thing to do these days’ inside pages of *Love Carefully*.^100^

As a finished product, shortcomings notwithstanding, the answer to these questions is yes. *Just Seventeen* was a hugely popular magazine and McFadyean a recognisable figure, signalling to teenage girls with her presence that the leaflet might be for them. Moreover, the production decisions borrowed heavily from teenage girls’ magazines in visual style, layout and content. The front cover of the pamphlet sported portraits of pop stars Dr Robert and Leslie & Shirlie, deployed as they would be in teen magazines, with short encouraging statements. The little smiling portrait of McFadyean is reminiscent of those which accompanied the problem pages she managed as Agony Aunt to *Just Seventeen.* All these layout features gave the impression that *Love Carefully* might be a couple of pages from a *Just Seventeen*, rather than a Brook production. Combined with the mix of medical and colloquial language in McFadyean’s introduction, added between the second draft and final production stages, the prose read like the health pages that were a weekly feature of *Just Seventeen*. The final familiar element was the cartoon strip. A mainstay of twentieth-century teen magazines, cartoon strips and photo stories was used to deliver romantic or comedic narratives and health messages in the pages of magazines like *MIZZ* and *Just Seventeen*.^101^ In combination, the signalling power of these trappings of feminine youth culture was such that in 1990, when the leaflet was considered for inclusion in the *Penis Pack*, a teaching pack on puberty designed for adolescent men with additional educational needs, it was suggested that the cartoon be removed from the context of the leaflet and used on its own, lest male teenagers be alienated by its femininity.^102^

Beyond this heavily edited inclusion in the *Penis Pack*, *Love Carefully* enjoyed wide circulation, appearing in a variety of sex education teaching packs and reading lists alongside other publications, as well as finding its way into libraries, youth groups and universities. Such was its popularity that it was reviewed for a second edition in 1990, again with Brook circulating it amongst interested members of the FPA for comment. Again, the term ‘AIDS virus’ was raised as an issue, as was Jim’s apparent reluctance to use condoms despite their growing popularity. As with the first edition, the pressure to produce a second edition quickly was emphasised, Brook gesturing to orders for the leaflets already waiting to be filled. Changes were made to the leaflet for the second edition—the language updated and McFadyean’s biography adjusted—but these changes were limited; Brook and other sex education NGOs instead relied on the use of *Love Carefully* in combination with other materials to mitigate any perceived shortcomings.

## Conclusions

Sex education and adolescent sexuality were topics of fraught and involved discussions among politicians, the media, and adolescents before the AIDS crisis, and the disease catalysed no immediate loosening of tongues on the subject of sex. Policy dictated how AIDS-related media intended for the consumption of children could frame the disease, dictating who could produce it, where such material could be used and by whom. Brook, like other NGOs involved in the fight against AIDS, was subject to these dictates from on high. Brook’s efforts to educate adolescents about AIDS, safer sex and consent were stymied by the bureaucratic politics and jurisdiction battles, which inhibited the government’s own response, while an awareness of what materials, ideas and information would not be allowed within the school gates encouraged the charity to omit explicit information on anything other than heterosexual intercourse. The charity thus anticipated objections from government, parents and governors and attempted to avoid the chagrin of these unintended audiences by avoiding controversial subjects and earmarking the text for use outside schools where the rules governing what teenagers could be told were less stringent. In creating a leaflet for teenagers on safer sex, Brook imagined a group of adolescents with agency: young people who, given the right information delivered in a respectful tone which valued their relationships, would practise safer sex. The group constructed by the leaflet presented an ideal of good behaviour where relationships were presented as ultimately caring, with strong female friendships guiding girls to challenge boys to use condoms, while male friendships were shown to support a caring performance of masculinity with condom use becoming an act of love and maturity.

The article began by situating Brook’s textual response to AIDS, *Love Carefully*, in the context of policy and practice, deploying techniques drawn from the history of emotions and literary analysis. By applying a Lacanian critique to both government sex education policy and the internal discussions of Brook and the FPA, in combination with the intertextual analysis of *Love Carefully*, this article revealed the ideological and emotional communities and contexts behind the text. This methodology could be applied to other didactic texts aimed at fostering positive health outcomes as a way of accessing how texts are changed by production context and authorial intent. Here the analytic traced the shared sense of anxiety, urgency, optimism and humour specific to the creation and content of *Love Carefully*, ultimately revealing it as representative of a genre without diminishing its unique context and content. These elements were demonstrated by following the *Love Carefully* leaflet through the drafting process, examining what was included and excluded from the final publication, anxiety about the inauthentic tone and worries about the framing of masculinity and condom use. This highlighted the problems that marred the production process and the awareness of those involved about the possibilities of misapprehension created by over simplification and misplaced emphasis.

When assessing the published document, *Love Carefully* provided an engaging and sophisticated text that aped the successful style of teenage magazines. As the article argued, the production of this varied and informed text was made possible by the supportive, if fractious, emotional community that Brook could draw on. This was a community with a widening pool of knowledge, expertise and content, equipped to produce *Love Carefully* as a salient document, familiar to teenagers in textual tone and visual design but novel enough to capture interest and deliver its messages of safer sex and consent. Brook and theFPA were a community speaking to, and for, teenagers, agitating for teenage empowerment and self-help through condom use but also dictating that safer sex was a fundamental aspect of care.

## Footnotes

2 Founded in 1963 by Helen Brook, The Brook Advisory Centre for Young People became known as Brook Advisory Centres, then The Brook as its services expanded and the number of centres grew. Currently, it is known as Brook, which is how I refer to it in this article unless quoting archival sources. ‘Brook | About Brook | History of Brook Timeline’, https://www.brook.org.uk/about-brook/history-of-brook-timeline, accessed 9 October 2017,; ‘Half a Century of Helpful People with Helpful Answers’, Wellcome Library, http://blog.wellcomelibrary.org/2014/07/half-a-century-of-helpful-people-with-helpful-answers/, accessed 9 October 2017.

3 McFadyean, *Love Carefully*, inside pages - see Figure 6

4 This charge is often evidenced by examinations of the passing of Section 28. For example, Jeffrey Weeks, *Sex, Politics and Society: The Regulation of Sexuality since* 1800 (London: Longman, 1989), 301–04; Jackie Stacey, ‘Promoting Normality: Section 28 and the Regulation of Sexuality’, in S. Franklin, C. Lury and J. Stacey, eds, *Off-Centre: Feminism and Cultural Studies* (London: Harper Collins, 1991), 284–304.

5 On AIDS-related ‘moral panics’ contemporary to *Love Carefully*, see Simon Watney, *Policing Desire: Pornography, AIDs and the Media* (London: Methuen, 1987), 41–46. On British AIDS policy led by biomedical elites and professionalised activists, see Virginia Berridge, *AIDS in the UK: The Making of Policy, 1981–1994* (Oxford: Oxford University Press, 1996).

6 Alison Hadley, ‘Press Release: *Love Carefully – Use a Condom*: Top-Ten Starts Endorse Brook Advisory Centres Leaflet for Young People’ (Brook Press and Information Office, 9 April 1987).

7 Across the 1980s and 1990s, teenage magazines played both an important role in the sex education of teenagers. E. Davies, ‘An Examination of Health Education in Teenage Magazines’, *Health Education Journal*, 1986, 45, 86–91; Kaye Wellings, *The Role of Teenage Magazines in the Sexual Health of Young People* (London: London School of Hygiene & Tropical Medicine: Department of Public Health and Policy, November 1996); Bridget Knight, ‘Teenage Magazines: Education or Titillation?’, in Pat Pinsent, ed., *Teenage Girls and Their Magazines* (London: National Centre for Research in Children’s Literature Roehampton Institute, 1998), 26–33.

8 Hadley, ‘Press Release’, 1.

9 See Deborah B. Gould, *Moving Politics: Emotion and ACT UP’s Fight Against AIDS* (Chicago: University of Chicago Press, 2009); Matt Cook, ‘“Archives of Feeling”: The AIDS Crisis in Britain 1987’, *History Workshop Journal*, 2017, 83, 51–78; Helen Coyle, ‘A Tale of One City: A History of HIV/AIDS Policy-Making in Edinburgh—1982–1994’ (unpublished PhD thesis, University of Edinburgh, 2008).

10 Dominick Lacapra, ‘Rethinking Intellectual History and Reading Texts’, *History and Theory*, 1980, 19, 245–76, 250.

11 While this approach bares similarity to Cook’s deployment of Cvetkovich’s conceptualisation of the emotional accumulation that occurs in cultural texts, the ‘archive of feeling’, my concentration on a single text and the didactic and ideological elements therein is methodologically more indebted to Bakhtin’s theories. Cook, ‘“Archives of Feeling”’, 51–78; Ann Cvetkovich, *An Archive of Feelings: Trauma, Sexuality, and Lesbian Public Cultures* (Durham, NC: Duke University Press Books, 2003).

12 See Deborah Lupton, *Moral Threats and Dangerous Desires: AIDS in the News Media* (London: Taylor & Francis, 1994); Virginia Berridge, *AIDS in the UK: The Making of Policy, 1981–1994* (Oxford: Oxford University Press, 1996); Simon Watney and Richard Bolton, *Policing Desire: Pornography, AIDS, and the Media*, new edition, 3rd, vol. 1 (Minneapolis: University of Minnesota Press, 1996); Jeffrey Weeks, *Sex, Politics and Society: The Regulations of Sexuality Since 1800* (New York: Routledge, 2014); Hannah Elizabeth, ‘[Re]Inventing Childhood in the Age of AIDS: The Representation of HIV Positive Identities to Children and Adolescents in Britain, 1983–1997’ (unpublished PhD thesis, University of Manchester, 2016).

13 Virginia Berridge, *AIDS in the UK: The Making of Policy, 1981–1994* (Oxford: Oxford University Press, 1996), 14–23.

14
*Ibid*., 14–23, 43–45.

15 Jeffrey Weeks *et al.*, ‘Community and Contracts: Tensions and Dilemmas in the Voluntary Sector Response to HIV and AIDS’, *Policy Studies*, 1996, 17, 108–10.

16 Herein lies a key difference between community activist groups that spoke ‘for and from a community’, such as the London Lighthouse and agencies which were ‘attempting to articulate the needs of a community’, which were unable to speak for themselves, like Brook and the FPA did on behalf of adolescents. See Weeks *et al.*, ‘Community and Contracts', 111–12.

17 Jonathan Zimmerman, *Too Hot to Handle: A Global History of Sex Education* (Princeton: Princeton University Press, 2015), 117–20; Lily Rothman, ‘How AIDS Changed the History of Sex Education’, *Time*, http://time.com/3578597/aids-sex-ed-history/, accessed 11 August 2017.

18 Anon, ‘Facts of Life: Sexual Attraction’, *Just Seventeen*, 17 November 1983; Elizabeth, ‘[Re]Inventing Childhood in the Age of AIDS’, 124–29.

19 Gillian Martins, ‘Facts of Life: Couples’, *Just Seventeen*, 15 December 1983, 1st edn; Melanie McFadyean, ‘Just Seventeen Advice’, *Just Seventeen*, 1 December 1983, 1st edon; Tricia Kreitman, ‘Body & Soul: We Are Not Able to Make Love’, *MIZZ*, 21 July 1985; Kate Charlesworth, ‘New Wives Tales’, *MIZZ*, 24 June 1985.

20 Key examples include the British Medical Association’s AIDS working group and the Sex Education Forum, founded in 1986. Elizabeth, ‘[Re]Inventing Childhood in the Age of AIDS’, 68–69.

21 Anon, ‘AIDS: The Facts’, *Just Seventeen*, 13 March 1985; Tricia Kreitman, ‘Can I Get AIDS from My Gay Friends? Body and Soul’, *MIZZ*, 12 April 1985.

22 Becky Field and Kaye Wellings, *Promoting Safer Sex: A History of the Health Education Authority’s Mass Media Campaigns on HIV, AIDS and Sexual Health 1987–1996* (London: Health Education Authority, 1997), 87–89.

23 Annie Franklin and Bob Franklin, ‘Growing Pains: The Development of the Children’s Rights Movement in the UK’, in Jane Pilcher and Stephen Wagg, eds, *Thatcher’s Children? Politics, Childhood and Society in the 1980s and 1990s* (London: Falmer Press, 1996), 94–113, 96–97.

24 John Kingdom, *No Such Thing as Society?: Individualism and Community* (Buckingham; Philadelphia: Open University Press, 1992), 2. For a more expansive discussion on this subject, see Elizabeth, ‘[Re]inventing Childhood in the Age of AIDS', 23–37.

25 Martin Durham, *Sex and Politics: Family and Morality in the Thatcher Years* (Basingstoke: Palgrave Macmillan, 1991), 13, 140.

26
*Ibid.*, 140–41.

27 Jane Pilcher, ‘Gillick and After: Children and Sex in the 1980s and 1990s’, in Jane Pilcher and Stephen Wagg, eds, *Thatcher’s Children? Politics, Childhood and Society in the 1980s and 1990s* (London: Falmer Press, 1996), 77–93; Debbie Epstein, Richard Johnson and Deborah Lynn Steinberg, ‘Twice Told Tales: Transformation, Recuperation and Emergence in the Age of Consent Debates 1998’, *Sexualities*, 2000, 3, 5–30.

28 Pilcher, ‘Gillick and After’, 78–79.

29 [1986] 1 FLR 229, [1985] UKHL 7, [1986] AC 112, http://www.bailii.org/uk/cases/UKHL/1985/7.html, accessed 18 August 2016.

30 House of Lords, ‘Gillick V West Norfolk and Wisbech Area Health Authority and Another’, *All England Law Reports*, 1985, 3, 402–37, see [1986] 1 FLR 229, [1985] UKHL 7, [1986] AC 112, http://www.bailii.org/uk/cases/UKHL/1985/7.html, accessed 18 August 2016; Pilcher, ‘Gillick and After’, 79–80.

31 [1986] 1 FLR 229, [1985] UKHL 7, [1986] AC 112, http://www.bailii.org/uk/cases/UKHL/1985/7.html, accessed 18 August 2016.

32 Pilcher, ‘Gillick and After’, 88–89.

33 The requirement that some of a school’s governors must be the parents of pupils attending the school was in proportion to the size of the school but always in greater numbers than were teacher governors. Education (No. 2) Act, (1986), 3–4, 24; Lynda Measor, Coralie Tiffin and Katrina Miller, *Young People's Views on Sex Education: Education, Attitudes and Behaviour* (London: Routledge, 2000), 19–20.

34 Ann Blair and Daniel Monk, ‘Sex Education and the Law in England and Wales: The Importance of Legal Narratives’, in Lutz D. H. Sauerteig and Roger Davidson, eds, *Shaping Sexual Knowledge: A Cultural History of Sex Education in Twentieth Century Europe* (London: Routledge, 2009), 37–51, 37.

35 Philip Meredith, *Sex Education: Political Issues in Britain and Europe* (London: Routledge, 1989), 10.

36
*Ibid*., 10.

38 Lesley Hall, ‘In Ignorance and in Knowledge: Reflections on the History of Sex Education in Britain’, in Sauerteig and Davis, eds, *Shaping Sexual Knowledge*, 19–36.

39
*Ibid*., 21.

40
*Ibid*., 21.

41 Elizabeth, ‘[Re]Inventing Childhood in the Age of AIDS’, 25.

42 Barbara H. Rosenwein, ‘Worrying about Emotions in History’, *The American Historical Review*, 2002, 107, 842.

43 Lacapra, ‘Rethinking Intellectual History and Reading Texts’, 250.

44 Mariela Vargova, ‘Dialogue, Pluralism, and Change: The Intertextual Constitution of Bakhtin, Kristeva, and Derrida’, *Res Publica*, 2007, 13, 415–40, 415; Mikhail Bakhtin, ‘Discourse in the Novel’, in Michael Holquist, ed., Caryl Emerson and Michael Holquist, trans, *The Dialogic Imagination* (Austin: University of Texas Press, 1981), 269–96.

45 ‘Background Notes For School Publications Unit Advisory Panel Meeting 11 March 1982’ (Brook, *n.d*.), SA.BRO.C.G.1.1 Schools Advisory Committee 1982–1989, The Wellcome Archive.

46 ‘Background Notes For School Publications Unit Advisory Panel Meeting 11 March 1982’.

47
*Ibid*.

48
*Ibid*.

49 ‘School Publications Advisory Panel Terms of Reference’, 1982, SA.BRO.C.G.1.1 Schools Advisory Committee 1982–1989, The Wellcome Archive.

50 ‘Terms of Reference’.

51
*Ibid*.

52 Helen Martins to Heads of Departments, ‘Re: Relations with Brook’, 5 July 1990, SA.FPA.C.G.4.6.18 [1 of 3], The Wellcome Archive.

53
*Ibid.*

54
*Ibid.*

55 Chris Pegler to Out-of-Schools Publication Group, ‘First Meeting of the Out-of-Schools Publication Group’, 2 July 1986, SA.BRO.C.G.2.1 Out-of-Schools Publication Group 1986–1996, The Wellcome Archive.

56 Martins to Heads of Departments, ‘Re: Relations with Brook’, 5 July 1990.

57
*Ibid*.

58 Jean Baugh, ‘Minutes of the Third Meeting of the Out-of-Schools Publications Group, 25 March 1987’, Minutes (Brook, 29 March 1987), SA.BRO.C.G.2.1 Out-of-Schools Publication Group 1986–1996, The Wellcome Archive.

59 Hadley, ‘Press Release’.

60 Elizabeth, ‘[Re]Inventing Childhood in the Age of AIDS’, 129–49.

61 For example, ‘AIDS myths’ were identified as a key obstacle to AIDS prevention by the BMA from 1986, leading them to flag this up in their evidence for the Social Services Committee on AIDS. BMA Working Party on AIDS, ‘Problems Associated with AIDS, Written Evidence for Submission to Social Services Committee: Provisional Outline of Issues to Be Covered’, 1986, B.43.1.1 1986–1988, WPA, BMA Archive.

62 Health Education Authority, ‘Initial AIDS Strategy’, 18 June 1987, FP2.2.1, The National Archive;

63
*Ibid*., p.7.

64 Hannah J. Elizabeth, ‘Getting around the Rules of Sex Education’, Wellcome Collection, 7 June 2018, https://wellcomecollection.org/articles/WxZnZyQAAPoF1PS8, accessed 20 March 2020.

65 Lynda Measor, Coralie Tiffin and Katrina Miller, *Young People’s Views on Sex Education: Education, Attitudes, and Behaviour* (New York: Routledge, 2000), 21–22; Jackie Green, ‘School Governors and Sex Education: An Analysis of Policies in Leeds’, *Health Education Journal*, 1994, 53, 40–51, 40.

66 Measor, Tiffin and Miller, *Young People’s Views on Sex Education*, 21–22.

67 Thomson, ‘Moral Rhetoric and Public Health Pragmatism’, 53.

68 Toni Belfield, ‘Comments on Draft 1 Love Carefully’, 29 January 1987, The Wellcome Archive.

69 Hadley, ‘Press Release’.

70 Baugh, ‘Minutes of the Third Meeting of the Out-of-Schools Publications Group, 25 March 1987’.

71 Helen Martins to FPA AIDS Working Group, ‘Memorandum: AIDS Leaflet’, 23 January 1987, SA.FPA.C.G.4.6.18 [2 of 3], The Wellcome Archive; Family Planning Information Service, ‘Minutes of Meeting between Alison Frater, Alison Hadley, Rosie and Helen Martins Regarding Love Carefully’, 3 March 1987, SA.FPA.C.G.4.6.18 [2 of 3], The Wellcome Archive.

72 Martins to Heads of Departments, ‘Re: Relations with Brook’, 5 July 1990.

74 I mention Service’s posts here because he was a powerful and knowledgeable lobbying force, with an intimate understanding of how health education policy was created and deployed by government. He cut his teeth in the policy arena during the 1967 campaign for abortion law reform, became Vice-Chair of the HEC in 1976, continuing in this post until the dissolution of the HEC in 1987 when he became Vice-Chair of the HEA. Health Education Council, ‘Health Education Council Members’ List’, 9 November 1985, FP1.16.1, The National Archive; Dilys Cossey, ‘Service, Alastair Stanley Douglas (1933–2013)’, www.Oxforddnb.Com/Index/101109742/Alastair-Service, *n.d*., accessed 23 August 2017; Anthony Grey, *Speaking Out: Writings on Sex, Law, Politics and Society, 1954–1995* (London: Bloomsbury Publishing, 1997), 196.

75 Belfield, ‘Comments on Draft 1 Love Carefully’.

76 Barbara Crowther, ‘The Partial Picture: Framing the Discourse of Sex in British Educative Films of the Early 1930s’, Sauerteig and Davidson, eds, *Shaping Sexual Knowledge*, 176–96, 178.

77 Belfield, ‘Comments on Draft 1 Love Carefully’.

78
*Ibid.*

79
*Ibid*.

80
*Ibid.*

81
*Ibid.*

82
*Ibid.*

83 This gendered division of responsibility, desire and sexual knowledge is fairly typical. In a survey of British sexual health education pamphlets and posters aimed at 13- to 19-year-olds produced between 1986 and 1996, Jewitt found that hegemonic conceptions of masculinity as sexually aggressive and femininity as sexually passive, though problematized, were typically reinforced. Carey Jewitt, ‘Images of Men: Male Sexuality in Sexual Health Leaflets and Posters for Young People’, *Sociological Research Online*, 1997, 2.

84 Another example used the tagline ‘AIDS. You know the risk. The decision is yours’. DHSS, ‘You Know What’s in His Mind but Do You Know What’s in His Blood?’, *MIZZ*, 28 February 1987; HEA, ‘“I Thought Only Gays and Drug Users Could Catch It”. That’s His Excuse, What Will Yours Be?’, 4 May 1988; Field and Wellings, *Promoting Safer Sex*, 14–17.

85 This eroticisation and celebration of the pleasurable possibilities of safer sex was increasingly prevalent in the health education produced by LGBTQ-focused charities in 1990s and 2000s. See, for example, Gay Men Fighting AIDS, *Semen Kit: Whenever You Dock Use Condoms & Lube*, 1994, The Wellcome Archive, https://wellcomecollection.org/works/m33njwx3, accessed 20 March 2020; Terrence Higgins Trust, *Positive or Negative, Safer Sex Is the Only Way*, 1990s, The Wellcome Archive, https://wellcomecollection.org/works/pmrvwxxb, accessed 20 March 2020.

86 For an extensive discussion of the FPA’s Men Too campaign, see Katherine Jones, ‘“Men Too”: Masculinities and Contraceptive Politics in Late Twentieth Century Britain’, *Contemporary British History*, 17 June 2019, 44–70.

87 Belfield, ‘Comments on Draft 1 Love Carefully’; Kaye Wellings, ‘Men Too--a Retrospective View of the Family Planning Association’s Male Responsibility Campaign’, *Planned Parenthood in Europe Regional Information Bulletin*, 1986, 15, 3–14.

88 FPA AIDS Working Group, ‘Comments on Draft 2 of Love Carefully’, 28 January 1987, SA.FPA.C.G.4.6.18 [2 of 3], The Wellcome Archive.

90
*Ibid*.

92 The pages of teenage magazines in the 1980s were littered with discussions of the ‘double standard’, wherein ‘boys can sleep around but girls are expected to be virgins’—this perception, it was argued, fostered the problems adolescent girls experienced around condom negotiation. See, for example, Suszie Hayman, ‘“What Boys Think” about Love, Sex and Birth Control’, *Just Seventeen*, 38 (6 March 1985), 18; Tricia Kreitman, ‘The Best Thing Anyone Told Me about Sex …’, *MIZZ*, 5 (21 June–5 July 1985), 16. See Elizabeth, ‘[Re]Inventing Childhood in the Age of AIDS’, 143–44.

93 Helen Martins, ‘Humorous Letter to Jane and Chris’, 1987, SA.FPA.C.G.4.6.18 [2 of 3], The Wellcome Archive; Chris Pegler and Jane Fraser, ‘Humorous Reply to Jane Martins’, 1987, SA.FPA.C.G.4.6.18 [2 of 3], The Wellcome Archive.

94 Belfield, ‘Comments on Draft 1 Love Carefully’; FPA AIDS Working Group, ‘Comments on Draft 2’.

95 Here I borrow Douglas’ description of the work-like function of jokes, Mary Douglas, *Implicit Meanings: Selected Essays in Anthropology*, 2nd edn (London: Routledge, 1999), 148.

96
*Ibid*., 152.

97 Elizabeth, ‘[Re]Inventing Childhood in the Age of AIDS’, 56–109.

99 Belfield, ‘Comments on Draft 1 Love Carefully’.

101 Angela McRobbie, *Feminism and Youth Culture: From ‘Jackie’ to ‘Just Seventeen’* (London: Macmillan Education Limited, 1991), 145–47; Charlesworth, ‘New Wives Tales’, 52–53.

102 Brook Advisory Centres Education and Publications Unit, ‘Model Penis Pack - Draft of Suggested Teaching and Presenters’ Notes’, June 1990, SA.BRO.C.G.2.1 Out-of-Schools Publication Group 1986–1996, The Wellcome Archive; Jane Fraser to All members of OSPG, ‘Penis Pack - Decisions for OSPG’, 21 July 1990, SA.BRO.C.G.2.1 Out-of-Schools Publication Group 1986–1996, The Wellcome Archive.

1 Melanie McFadyean, *Love Carefully: Use a Condom* (Brook AIDS Concern, 1987), EPH 509 AIDS, The Wellcome Archive.

37 Rachel Thomson, ‘Moral Rhetoric and Public Health Pragmatism: The Recent Politics of Sex Education’, *Feminist Review*, 48, 1, 40–60, 49–50.

73 Belfield, ‘Comments on Draft 1 Love Carefully’.

89
*Ibid*.

91 FPA AIDS Working Group, ‘Comments on Draft 2 of Love Carefully’, Cartoon.

98 McFadyean, ‘Love Carefully’, front and back.

100 McFadyean, inside pages.

## Notes

Hannah J. Elizabeth is a cultural historian specialising in the British history of HIV, sexuality, public health, emotion, childhood and children’s media working as a research fellow on the Wellcome funded project 'The Cultural History of The NHS’ University of Warwick. Their PhD investigated the history of HIV/AIDS-related media produced for the consumption of children and young adults in Britain from 1981 to 1997.

